# Sclerosing stromal tumor of the ovary: a case series and review of literature

**DOI:** 10.1259/bjrcr.20210155

**Published:** 2021-12-03

**Authors:** Christof Mittermair, Teresa Margarida Cunha, Romana Urbas, Horst Koch, Rosemarie Forstner

**Affiliations:** 1 Department of Radiology, Paracelsus Medical University, Salzburg, Austria; 2 Department of Radiology, Instituto Portugues de Oncologia de Lisboa – IPOLFG, Lisbon, Portugal; 3 Department of Pathology, Paracelsus Medical University, Salzburg, Austria; 4 Department of Gynecology, Paracelsus Medical University, Salzburg, Austria

## Abstract

Sclerosing stromal tumor of the ovary is a rare benign sex-cord stromal tumor that affects primarily young females. In a series of six patients (mean 24,6, median 19 years) findings of six MRIs and one CT were analyzed. Unilateral tumors ranging from 6 to 8 cm were found in all patients. The tumors were well encapsulated and polylobulated. The morphology was mixed solid and cystic in three and solid in three patients. In CT, a hypervascular tumor with centripetal enhancement was seen. In MRI *T*_2_ weighted imaging showed low signal intensity of the solid tissue in all cases and low diffusion-weighted imaging signal of the solid tissue in high b-value diffusion-weighted imaging in three patients. Contrast enhancement was avid with extension from the periphery in all patients. Knowledge of these distinct radiological features of sclerosing stromal tumor is important, as in the Ovarian-Adnexal Reporting and Data System risk classification system this may be scored as Ovarian-Adnexal Reporting and Data System 5. Because of its non-aggressive clinical course, pre-operative imaging assists to avoid unnecessary extensive surgery and to preserve the patient’s fertility by only resecting the tumor and preserving the ovary. Sclerosing stromal tumor of the ovary presents pathognomonic features in MRI that allow a specific pre-operative diagnosis and selecting candidates for fertility-sparing surgery.

## Introduction

Sclerosing stromal tumor is a rare benign sex-cord stromal tumor of the ovary that affects primarily young females under the age of 30.^1,2^ It accounts for less than 5% of all ovarian sex-cord stromal tumors^2^ and was first described by Chalvarijan and Scully in 1973.^3^ Patients usually present with menstrual irregularities^4^ and/or abdominal pain.^2^ Ascites is only found in a minority of cases.^5^ It has distinct radiological features and a non-aggressive clinical course. No malignant forms have been reported in the literature so far and surgical resection is the treatment of choice.^2,6^

Ultrasound findings are usually non-specific and may overlap with those of malignant ovarian tumors, therefore further imaging evaluation with CT or MRI is usually required.^2^

Most published literature on sclerosing stromal tumors of the ovary focuses mainly on the histological and immunohistochemical features of this tumor entity,^7,8^ and only a few published articles focus on its imaging findings, particularly on CT and MRI correlation.^1,5,6^

Furthermore, to our knowledge our series of six cases is among the largest series described in the literature.^9^

## Case report

A 21-year-old female nullipara patient was referred to our hospital in Salzburg by her gynecologist for an incidentally detected adnexal mass on a routine examination. The patient denied any abdominal pain or menstrual irregularities. Transvaginal ultrasound depicted an adnexal mass arising from the right ovary, with mixed solid and cystic components, measuring 5,5 × 4,6 cm. Free fluid was seen in the Douglas cul-de-sac. β-HCG was negative (<1 U l^−1^) and no signs of inflammation were present at admission. Tumor markers were also negative (carcinoembryonic antigen = 0,6 µg l^−1^ and CA 12–5 = 18 U ml^−1^).

Hormone tests were also performed, and a slight increase in testosterone was detected (0,65 ng ml^−1^) (normal values range between 0,08 and 0,48). Follicle-stimulating hormone, Luteinizing hormone, prolactin, progesterone and estradiol E2 were within normal ranges.

**Figure 1.**
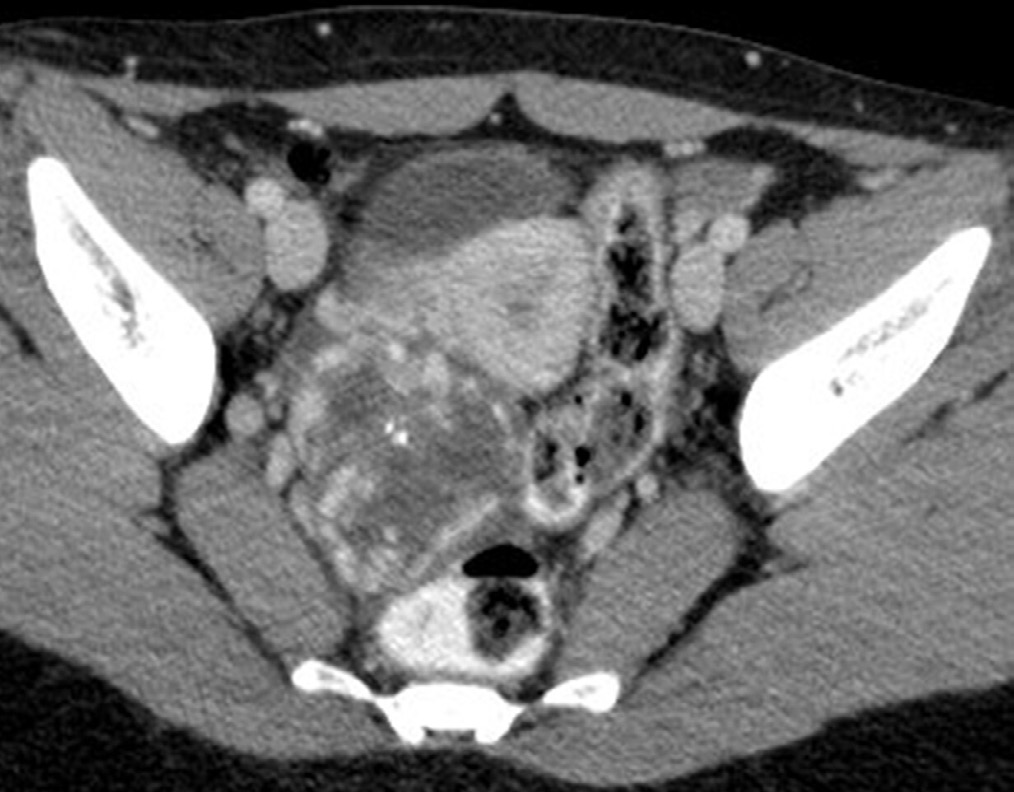
Axial contrast-enhanced CT shows a 6 cm mass in the right adnexal region with centrally located calcifications, a central cystic component and avid contrast peripheral enhancement.

CT in the portal venous phase showed a 6 cm polylobulated mass of the right ovary with intralesional calcifications, a central cystic component and avid peripheral contrast enhancement (Figure 1). The left ovary, uterus and rectum/sigmoid colon were slightly displaced by the tumor but were otherwise unremarkable. No pathological lymph nodes or distant metastases could be detected. Only a small amount of free fluid (<1 cm) was present.

**Figure 2.**
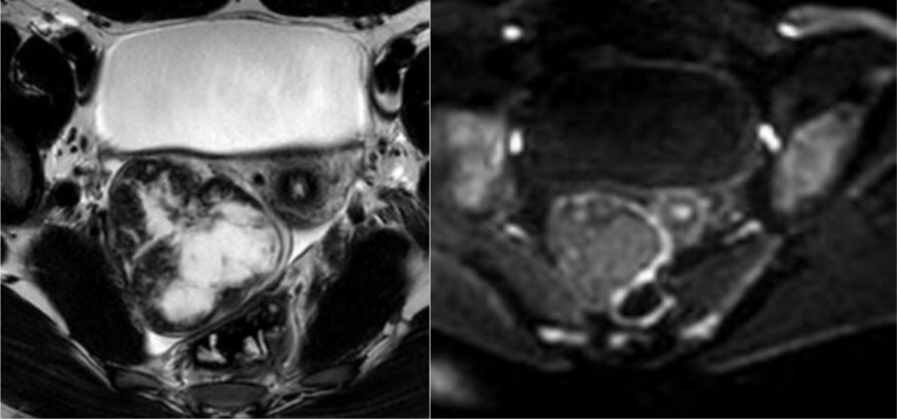
Axial *T*_2_ weighted and diffusion-weighted MRI (b-value = 1200 s/mm^2^) shows a heterogenous solid mass with a cystic central area, hypointense septa, hypointense capsule and no areas of restricted diffusion.

**Figure 3.**
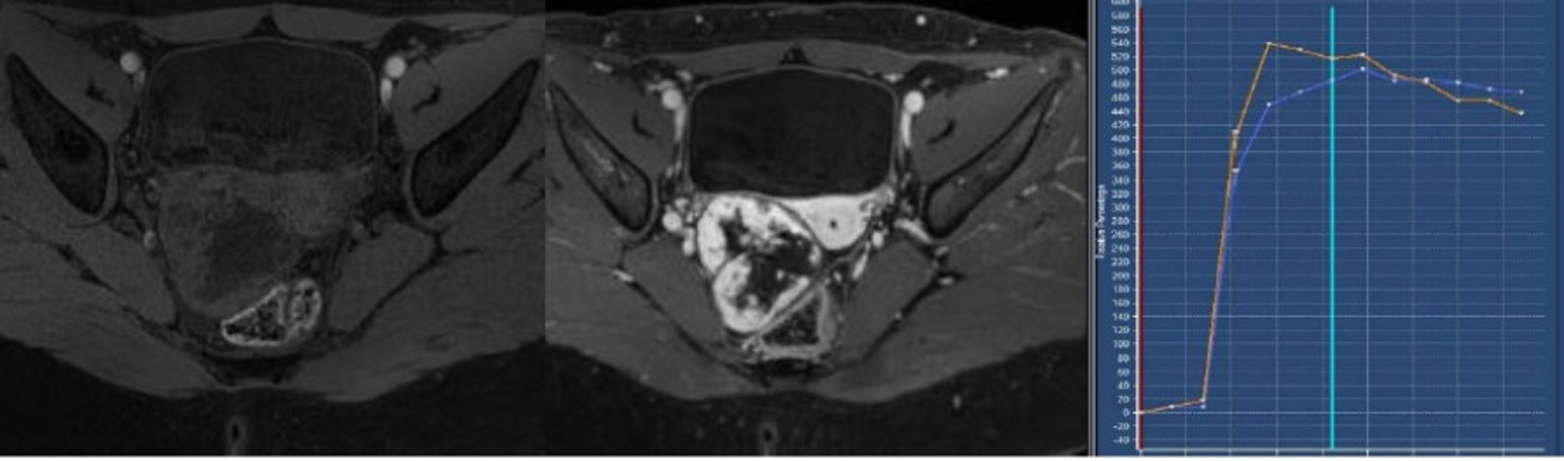
Axial *T*_1_ weighted image with fat suppression before and after intravenous contrast injection showing no evidence of internal hemorrhage and a Type 3 intensity curve in the periphery of the tumor that is earlier and higher than the enhancement of the outer myometrium (orange *vs* blue graph).

Further work-up with MRI including dynamic contrast-enhanced sequences was performed (Figure 2). *T*_2_ weighted images (*T*_2_WI) showed a heterogenous hypointense solid tumor with a hyperintense central region and a hypointense capsule. No intralesional hemorrhage or areas of restricted diffusion were found. After intravenous contrast injection, the lesion showed early and avid peripheral contrast enhancement with centripetal progression on the following sequences. Time–intensity curve demonstrated enhancement higher than myometrium (Figure 3). The normal ovarian parenchyma was compressed laterally. No infiltration of other organs, such as the rectum/sigmoid colon, could be detected. The uterus and the left ovary were unremarkable as well.

Laparoscopic adnexectomy of the right ovary was performed and further histological and immunohistochemical examination confirmed the diagnosis of a sclerosing stromal tumor of the right ovary. Immunohistochemically, the sample showed typical positivity for Inhibin where else focal expression of Desmin could, among others, not be detected. No malignant cells could be detected in the sample. The patient was discharged clinically well 2 days after the surgery. Follow-up with ultrasound within 1 year was recommended.

### Comparison with five other cases of sclerosing stromal tumor of the ovary at our institutions

At our departments in Salzburg and Lisbon, six cases of this tumor entity were observed in the last few years.

All patients were aged between 14 and 33-years-old (mean 24,6 years, median 19 years) and had a unilateral tumor ranging between 6 and 8 cm in size (mean 7 cm, median 7 cm).

Three tumors originated from the left ovary and the others from the right ovary (Table 1).

**Table 1.** The six cases of sclerosing stromal tumors in detail

	Age in years	Side	Size in cm	Morphology	Polylobulated and thin capsule	Contrast enhancement	Signal of solid parts on *T*_2_WI and DWI (*b* > 800)		Pelvic fluid
Case 1^10^	14	Left	8	Solid	Yes	Avid	Low		<1 cm
Case 2	15	Left	7	Solid-cystic	Yes	Avid	Low		>1 cm
Case 3	18	Left	7	Solid	Yes	Avid	Low	Low	No
Case 4^11^	20	Right	8	Solid	Yes	Avid	Low	Low	<1 cm
Case 5	21	Right	6	Solid-cystic	Yes	Avid	Low	Low	<1 cm
Case 6	33	Right	6	Solid-cystic	Yes	Avid	Low		No

MRI protocol included *T*_1_- and *T*_2_ weighted sequences in multiple planes as well as *T*_1_ weighted sequences with fat-suppression after intravenous contrast injection. In three cases, diffusion-weighted imaging (DWI) was also performed.

In all cases, tumors were polylobulated, had a well-defined capsule and showed avid contrast enhancement. Three of these tumors were mixed solid-cystic and three tumors were purely solid. Only in one case (16,6 %), pathological amount of free fluid was seen in the Douglas cul-de-sac

### Pathology

**Figure 4.**
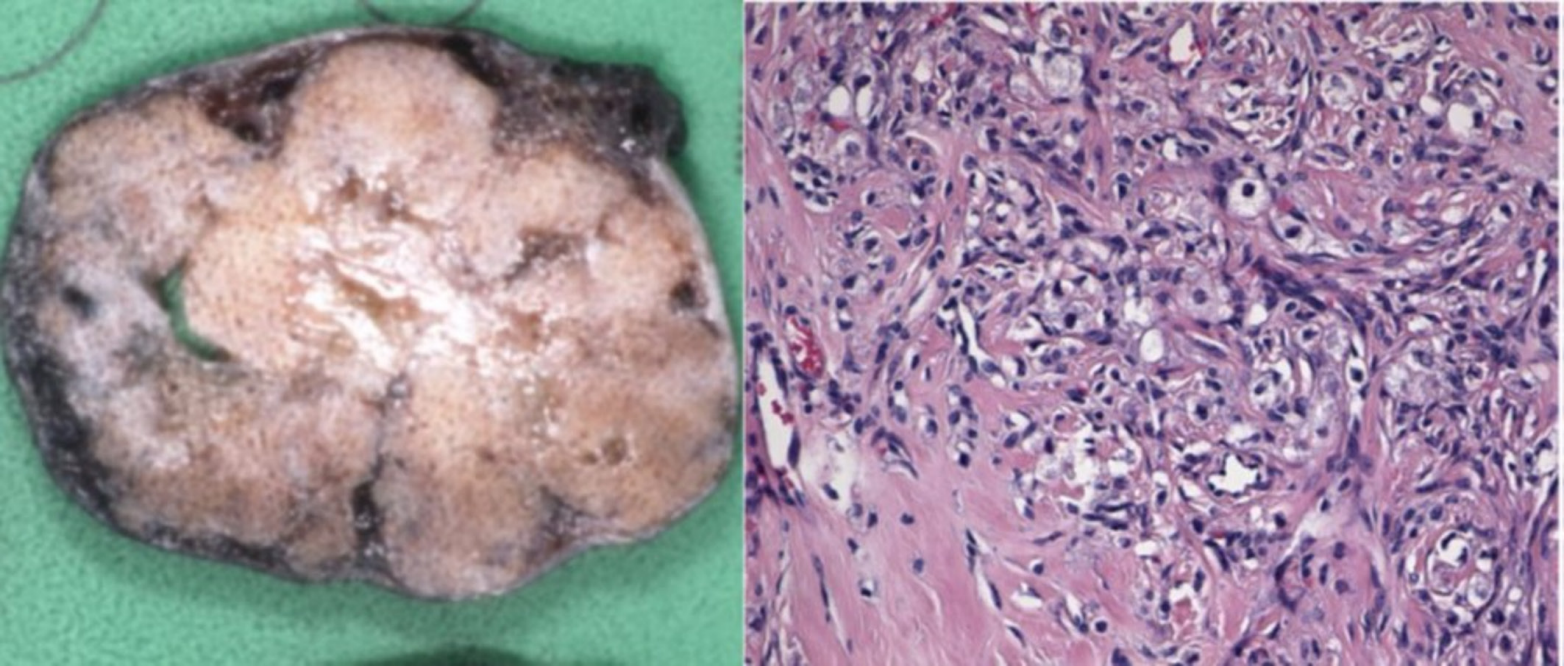
Pathologic specimen showing a mixture of cellular areas, cystic areas as well as densely packed myxoid and collagenous tissue.

In the literature, typical pathologic features are described. This tumor entity often shows a mixture of cellular and cystic areas, as well as densely packed myxoid and collagenous tissue (Figure 4). Enlarged vessels can be found in between these elements. Edematous ovarian cortical stroma usually surrounds the tumor. This combination of different types of tissue in the tumor is called a “pseudolobular pattern” resulting from alternating cellular and hypocellular areas and correlates with the typical imaging features on *T*_2_ weighted images and the avid enhancement of contrast^1,12^ (Table 2). Expression of Inhibin as it is in our patient is among others also often described in literature.^13^

**Table 2.** CT and MRI features of sclerosing stromal tumor of the ovary. ^2,5^

Age	Females under the age of 30
Morphologic and structural features	Polylobulated tumor Inhomogeneous *T*_2_WI and DWI hypointense solid parts Solid-cystic or solid Centrally located intralesional calcifications
Contrast enhancement	Avid contrast enhancement from the periphery to the center
Other features	Most often unilateral, rarely bilateral Rarely associated with ascites

DWI, diffusion-weighted imaging; *T*_2_WI, *T*_2_ weighted imaging.

## Discussion

Sclerosing stromal tumor of the ovary represents an extremely rare subtype of sex cord stromal tumors in young females, typically seen during the second and third decades. In contrast, the more common benign sex cord stromal tumors (fibroma and thecoma) are uncommonly encountered in this age. Patients usually present with menstrual irregularities^4^ and/or abdominal pain.^2^ The tumor is most likely hormone-inactive, although in some cases, androgenic effects such as virilism were described.^2^

Due to their solid or solid-cystic morphology, ultrasonographic findings may mimic a malignant ovarian tumor.^2^ Thus, further imaging evaluation, preferably with MRI, is warranted to pre-operatively correctly diagnose this benign tumor entity that has pathognomonic imaging features.

In the literature, only about 200 cases have been described so far.^9^

To our knowledge, our series of six cases is one of the largest series described so far,^9,13,14^ with only a few of them focusing on the imaging features of this tumor entity.

In the existing publications, this tumor is usually unilateral and described as a heterogenous solid tumor with septa and cystic areas (described as a “spoke-wheel pattern”), inhomogeneous *T*_2_WI hypointense solid parts, a thin capsule and early avid contrast enhancement extending from the periphery to the center (Table 2). This centripetal enhancement pattern has been described as hemangioma-like and was seen in all our above-mentioned cases.^2,4^

Our series also includes the DWI findings in three cases, with low signal intensity (SI) on high *b*-values, due to its benign stromal nature. In other case-reports that included DWI, no areas of restriction were also found.^15–17^ According to the recently published O-RADS MRI risk classification system, Class 2 lesions (likely benign) also include solid tumors with unique features of low SI on *T*_2_WI and on DWI, frequently related to ovarian fibromas and thecomas. Sclerosing stromal tumors do not classify as O-RADS 2 lesions as they demonstrate inhomogeneous hypointense signal on *T*_2_WI. Furthermore, due to their contrast enhancement (higher than that of the myometrium) they have to be classified as O-RADS 5, which is associated with a high risk of malignancy (positive-predictive value 90%). However, in addition to assigning a score predictive for malignancy, O-RADS acknowledges that the final diagnosis may be suggested in cases of classical imaging findings (*e.g.* lymphoma, granulosa cell tumors, peritoneal pseudocysts etc.). We consider that sclerosing stromal tumors could be included in this list.^18^

Because this is a benign tumor with a non-aggressive clinical course, the primary aim of pre-operative imaging is to avoid unnecessary extensive surgery and to preserve the patient’s fertility by only resecting the tumor and preserving the ovary.^19^

The differential diagnosis of sclerosing stromal tumors of the ovary in young females should include other sex cord stromal tumors such as thecomas, malignant germ cell and epithelial tumors, lymphoma and metastases^6,11,20,21^ (Table 3). Like in our cases, these differential diagnoses can usually be excluded due to the characteristic imaging findings of this tumor entity in CT and MRI.

**Table 3.** Differential diagnostic features of solid ovarian tumors in pre-menopausal women^20,21^

	Age (yrs)	Laterality	CT	MRI	Typical or additional features
**Sclerosing stromal tumor**	<30	Unilateral	Solid with cystic areas, avid contrast uptake	Low on T2 and DWI*, avid contrast uptake	Rim like contrast enhancement
**Thecoma**	All ages	Unilateral	Homogenous, delayed enhancement	Low on T2 and DWI*, typically hypovascular	
**Dysgermin-oma**	20–30	Unilateral	Multinodular, enhancing septa, speckled calcifications	Intermediate on DWI*, high SI on DWI, enhancing septa	Often large, serum LDH elevation
**Granulosa cell tumor**	<30	Unilateral	Variable from solid to purely cystic	Intermediate on T2, high SI on DWI*, intermediate to high contrast uptake	Sponge like appearance, estrogenic effects
**Immature teratoma**	<20	Unilateral	Predominantly solid, large, heterogenous, small foci of fat and scattered calcifications	Tiny areas of high SI on T1 in a solid mass, high on DWI*	Serum AFP elevation, may coexist with benign teratoma
**Ovarian cancer**	>20	Uni- or bilateral	Solid or cystic and solid	Intermediate on T2, high SI on DWI*, intermediate to high contrast uptake	Borderline tumors may precede, familial predisposition
**Metastases**	Premenopausal	Often bilateral	Multinodular surface, minimal to moderate enhancement	Intermediate on T2, high SI on DWI*, intermediate to high contrast uptake	Breast or stomach cancer, often small
**Lymphoma**	All ages	Uni- or bilateral	Well defined, hypovascular	Homogeneous, low to intermediate SI on T2, high SI on DWI*, low ADC	Mostly B cell lymphoma, extremely rare, lymphadenopathy

ADC, apparent diffusion coefficient; AFP, alpha fetoprotein; DWI, diffusion-weighted imaging; LDH, lactate dehydrogenase; SI, signal intensity.

DWI*: using high b-value (800–1000 s/mm2).

Due to the young age of these patients, and in order to avoid unnecessary radiation exposure, MRI is the preferred imaging modality.

## Conclusion

Sclerosing stromal tumor is a rare benign sex-cord stromal tumor of the ovary affecting mainly young females displaying characteristic imaging features on MRI and CT.^2^ Because of its benign nature, pre-operative diagnosis with CT or MRI is pivotal in order to avoid extensive surgery and preserve patient’s fertility.^19^

## Learning points

- Sclerosing stromal tumor is a rare benign sex-cord stromal tumor of the ovary affecting mainly young females displaying characteristic imaging features on MRI and CT.
- This tumor is usually unilateral, a heterogenous solid tumor with septa and cystic areas (“spoke-wheel pattern”), inhomogeneous *T*_2_WI hypointense solid parts and early avid contrast enhancement extending from the periphery to the center (hemangioma-like enhancement pattern).
- Because of its benign nature, preoperative diagnosis with CT or MRI is pivotal to avoid extensive surgery and preserve patient’s fertility.
